# DNA-Aptamers Binding Aminoglycoside Antibiotics

**DOI:** 10.3390/s140203737

**Published:** 2014-02-21

**Authors:** Nadia Nikolaus, Beate Strehlitz

**Affiliations:** Helmholtz Centre for Environmental Research (UFZ), Permoserstraβe 15, Leipzig 04318, Germany; E-Mail: nadia.nikolaus@ufz.de

**Keywords:** aptamer, Capture-SELEX, aminoglycoside antibiotic, kanamycin

## Abstract

Aptamers are short, single stranded DNA or RNA oligonucleotides that are able to bind specifically and with high affinity to their non-nucleic acid target molecules. This binding reaction enables their application as biorecognition elements in biosensors and assays. As antibiotic residues pose a problem contributing to the emergence of antibiotic-resistant pathogens and thereby reducing the effectiveness of the drug to fight human infections, we selected aptamers targeted against the aminoglycoside antibiotic kanamycin A with the aim of constructing a robust and functional assay that can be used for water analysis. With this work we show that aptamers that were derived from a Capture-SELEX procedure targeting against kanamycin A also display binding to related aminoglycoside antibiotics. The binding patterns differ among all tested aptamers so that there are highly substance specific aptamers and more group specific aptamers binding to a different variety of aminoglycoside antibiotics. Also the region of the aminoglycoside antibiotics responsible for aptamer binding can be estimated. Affinities of the different aptamers for their target substance, kanamycin A, are measured with different approaches and are in the micromolar range. Finally, the proof of principle of an assay for detection of kanamycin A in a real water sample is given.

## 1. Introduction

Aptamers are short, single stranded DNA or RNA oligonucleotides that are able to bind specifically and with an affinity that is comparable to that of antibodies to their non-nucleic acid target molecules. Compared to antibodies, aptamers, particularly DNA ones, are more stable concerning degradation or denaturation and they are able to recognize a distinct epitope of a target molecule [1]. Binding between aptamer and target molecule is provided by different intermolecular interactions like electrostatic interactions between charged groups, stacking of aromatic structures contained in organic compounds and the nucleobases, hydrogen bonds, and the complementary in three-dimensional shape. Aptamers can be developed for a vast variety of possible targets ranging from small organic molecules over peptides and proteins to complex structures like cells or viruses [2]. Since the first development of aptamers in 1990 [3], these antibody rivaling structures are on their way to finding applications not only as receptors in medical and environmental analysis or the analysis of food and pharmaceuticals, but also for application as new drugs in medicine or for clinical diagnostics imaging and some others. Aptamers are usually generated by an iterative *in vitro* process called SELEX that constricts a starting library consisting of a multitude of random nucleic acid molecules to a small subset of strongly binding species by successive rounds of binding to the target, elution of bound oligonucleotides and their amplification [4]. Selection can be modified by introduction of negative or counter selection steps in order to increase the stringency of binding conditions or to obtain aptamers binding to specific epitopes in the target molecule or even to distinguish between chiral molecules [2,5].

In case of an enrichment of binding oligonucleotides, a pool of aptamers with unknown sequence will be obtained. In order to get individual aptamers, this pool has to be cloned and the sequence has to be determined. After sequencing, the now known sequences can be analyzed according to consensus motifs or conserved regions within the sequences which indicate possible binding regions. A consecutive step would be the testing of single aptamers in order to choose those with the best binding characteristics for the envisaged application. Also possible modifications for specific needs, like immobilization or detection paths, can be inserted.

The possible application of DNA aptamers in biosensors for the detection of environmental contaminants or in materials for their enrichment or filtration is a relatively new field of application. The pollution of waters—surface water, ground water, drinking and waste water—by pharmaceutical residues is a serious problem. Those residues find their way into the water cycle after administration of the drugs via human or animal excretion. They are often relatively stable, some of them are resistant to degradation in sewage plants and are found, even though in trace concentrations, in all environmental compartments. Antibiotic residues represent a special problem because they contribute to the emergence of antibiotic-resistant pathogens and reduce the effectiveness of the antibiotic to combat human infections.

Aminoglycoside antibiotics were discovered in the 1940s and are to date the most commonly used antibiotics worldwide thanks to the combination of their high efficacy with low cost even though they have serious side effects of renal and auditory toxicity. Aminoglycoside antibiotics are low-molecular-weight molecules of approximately 300–600 Daltons. All natural and semisynthetic aminoglycosides share a similar structure consisting of several, usually three, rings. These rings are cyclitols (a saturated 6-carbon ring structure) and five or six-membered sugars that are linked via glycosidic bonds. Aminoglycoside antibiotics have a broad antibacterial spectrum and they are effective against gram-negative bacteria. They show bactericidal properties, i.e., they are able to kill bacteria and not only to prevent their growth [6].

Misuse or overuse of antibiotics in general both in human as in veterinary medicine as well as the use of antibiotics as growth enhancers in livestock create selective evolutionary pressure that enables antimicrobial resistant bacteria to survive and propagate preferentially. Injudicious subtherapeutic use of medically important antimicrobial drugs in food-producing animals for production or growth-enhancing purposes seriously adds to the problem of resistance formation [7]. Kallova *et al.* found the dramatically increase of the aminoglycoside resistance in clinical Gram-negative bacterial isolates in Slovakia within ten years [8]. They determined the importance and disseminations of enzymatic mechanisms for this resistance. Molecular mechanisms for resistance formation concerning aminoglycoside antibiotics are either the modification of the antibiotic targets, that is the bacterial ribosomal rRNA [9] or enzymatic modification of the antibiotic drug itself, resulting in a product that is no longer effective as antibiotic [10].

Although the European Union banned the subtherapeutic feeding of antibiotics and related drugs to food-producing animals in 2006, the amount of antibiotics released in the environment from farms and human's sewage will likely stay at rather high levels in the future. This means that besides control policies in the use of antibiotics, studies for improving their degradation are needed [11]. The development of analytic tools for fast and easy detection and new materials to remove antibiotic drugs from the environment would be very desirable. The aim of this study was to characterize and modify DNA aptamers for kanamycin A that were developed beforehand by Capture-SELEX [12] regarding specificity and affinity. Also some truncated variants of these aptamers were tested. Moreover, the proof of principle of an assay was tested for the detection of kanamycin A in real effluent samples of a water treatment plant.

## 2. Experimental Section

### 2.1. Chemicals

**Figure 1.**
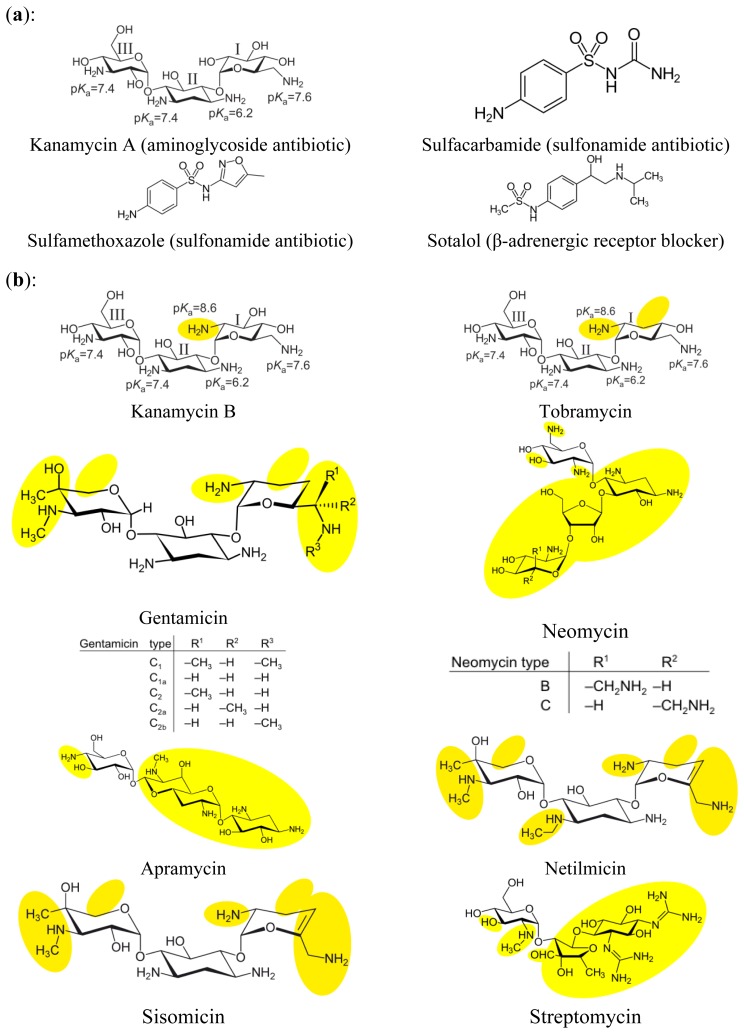

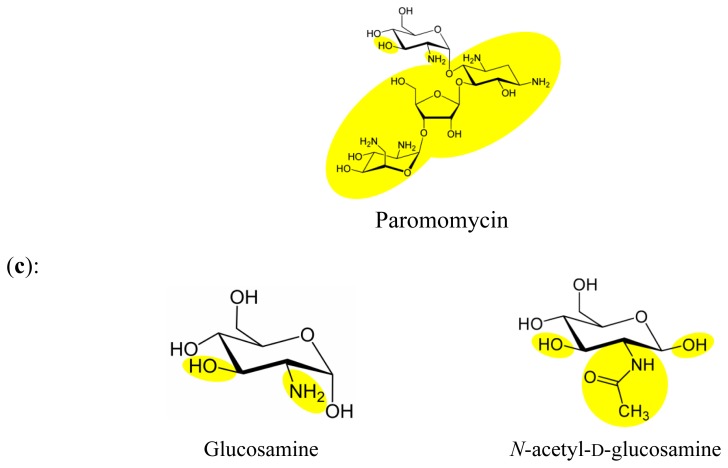
(**a**) Chemical structures of the selection targets. Numbering of the rings and p*K*_a_ values of amino groups according to [13]; (**b**) Chemical structures of aminoglycoside antibiotics other than kanamycin A. Differences in the structure compared to kanamycin A are marked in yellow. Numbering of the rings and p*K*_a_ values of amino groups according to [13]; (**c**) Chemical structures of amino sugars. Differences in the structure compared to kanamycin A are marked in yellow.

All chemicals for preparing buffers and solutions were obtained from Merck (Darmstadt, Germany). Kanamycin A disulfate salt dihydrate, gentamicin sulfate salt hydrate, glucosamine, *N*-acetyl-D-glucosamine, neomycin trisulfate hydrate, sisomicin sulfate salt, streptomycin sulfate salt, sulfacarbamide, sulfamethoxazole, sotalol hydrochloride, and tobramycin were purchased from Sigma-Aldrich (Seelze, Germany). Kanamycin B and netilmicin sulfate were purchased from LKT Laboratories (St Paul, MN, USA), paromomycin sulfate was purchased from U.S. Pharmacopeia (Rockville, MD, USA), and apramycin sulfate from Applichem (Darmstadt, Germany). For an overview of the pharmaceuticals and their chemical structures see Figure 1. Four of the pharmaceuticals (kanamycin A disulfate salt dihydrate, sulfacarbamide, sulfamethoxazole, and sotalol hydrochloride), were used as aptamer selection target mixture. Individual stock solutions of the pharmaceuticals were prepared, diluted in selection buffer and mixed to the final concentration of 1 mmol L^−1^ for each substance. The pH value was adjusted to ∼7.6 and the mixture was sterile-filtered using a syringe filter with the pore size of 0.22 μm (VWR, Dresden, Germany), aliquoted and stored at −18 °C.

### 2.2. Selection and Truncation of Aptamers

The aptamers used here were obtained by the Capture-SELEX procedure. The method as well as the development of the aptamers was described in detail earlier [12]. The peculiarity of the Capture-SELEX is that the oligonucleotides of the random library are immobilized on magnetic beads, whereas the target molecules can be used in solution. By this way, aptamer selection is possible for small molecules which are not immobilizable. Briefly, the oligonucleotides of the random library contain a modified randomized region including 12 nucleotides with fixed sequence, the docking sequence (TGAGGCTCGATC) and two random regions of different length (N10 and N40), and the primer binding sites at both ends (5′primer: ATACCAGCTTATTCAATT, 3′primer: AGATAGTAAGTGCAATCT). The complete sequence of the oligonucleotides of the random library (BANK-S4) looks as follows: 5′ATACCAGCTTATTCAATT—N10—TGAGGCTCGATC—N40 —AGATAGTAAGTGCAATCT 3'. The 18 nt-sequences at the 5′and 3′ends, respectively, are primer binding sites that serve for the amplification of the obtained oligonucleotides in a PCR. The capture oligo iODN2Sp (5′Bio-GTC-HEGL-GATCGAGCCTCA 3') contains the complementary sequence of the docking sequence and a hexaethylene glycol spacer (HEGL) near the 5'-end. This capture sequence is immobilized via its 5′biotin label on superparamagnetic beads (Superparamagnetic Dynabeads^®^ M-270 Streptavidin, diameter: 2.8 μm). The library oligonucleotides were anchored to the beads by hybridization of the docking sequence with the capture sequence. After several wash steps and steps to eliminate loosely attached sequences, the selection target mixture was added to the solution. In order to bind to specific target molecules, the oligonucleotides have to undergo a structure switch from hybridization with the capture oligo to the three-dimensional structure that allows them to connect with its target. Those target binding oligonucleotides were multiplied and fluorescein (FAM) labeled at their 5′ends in a PCR reaction and then used as the starting set of sequences for the next round of Capture-SELEX.

**Figure 2.**
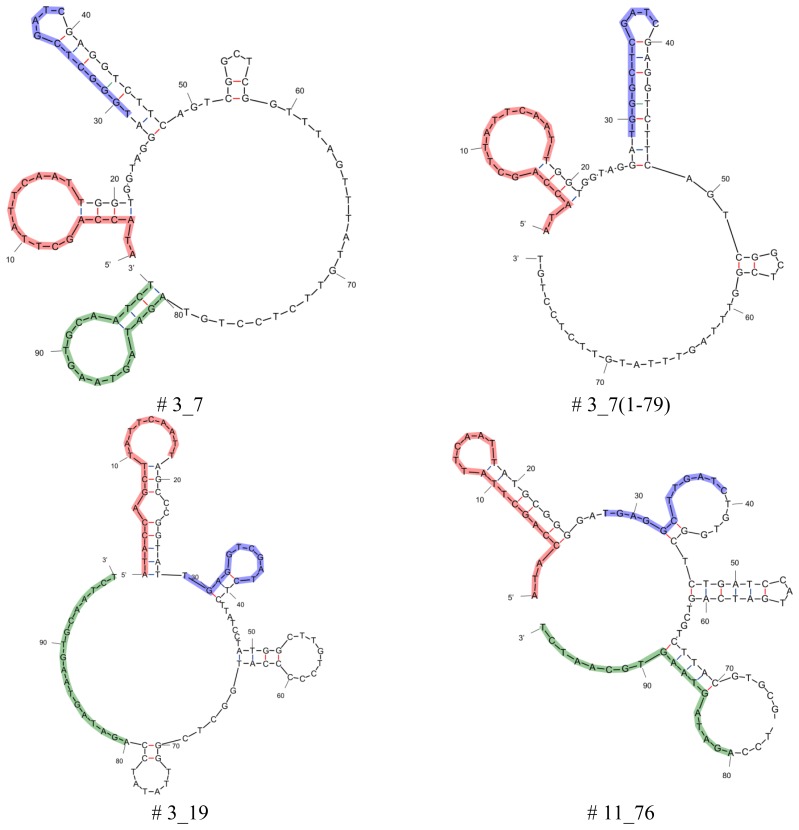

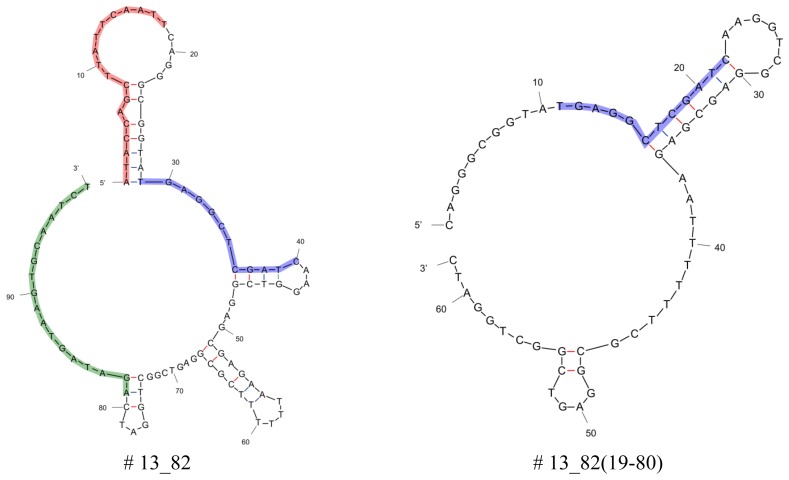
Secondary structures of selected aptamers and truncations. The 5′and 3′primer binding sites are highlighted in red and green, respectively. The docking sequence is highlighted in blue.

Aptamers that were obtained after 13 rounds of Capture-SELEX were analyzed for selectivity and affinity. Truncations of the best binding aptamers (aptamers # 3_7, # 13_82, # 11_76, and # 3_19) were performed by cutting the 5', the 3', or both primer binding sites and all twelve resulting truncated aptamers were analyzed in further specificity and affinity tests (data not shown). Aptamers or their truncations that proved to be binders with the highest affinity or specificity were selected and used here: aptamers # 3_19, # 3_7, # 11_76, and # 13_82; truncations # 3_7(1-79) lacking the 3′primer binding site and # 13_82(19-80) lacking both primer binding sites (see Table 1, for secondary structures see Figure 2).

Secondary structure analysis of selected aptamers and truncations was performed by means of the free-energy minimization algorithm using the web based tool mfold with salt correction [14,15] (Figure 2). All aptamers and the capture oligo iODN2Sp with different modifications were synthesized and PAGE purified by Microsynth (Balgach, Switzerland).

### 2.3. Specificity and Affinity Tests

Affinity and specificity tests were performed in triplicate either in bead based (“bead assay”) or microplate based assays (“MTP assay”) by fluorescence detection of the 5'-FAM labeled aptamers. Further affinity tests were performed using a surface plasmon resonance (SPR) system (Biacore X 100, GE Healthcare, Freiburg, Germany). The test formats will be described in more detail in the following sections.

#### 2.3.1. Fluorescence Detection

##### Bead Assay

**Figure 3.**
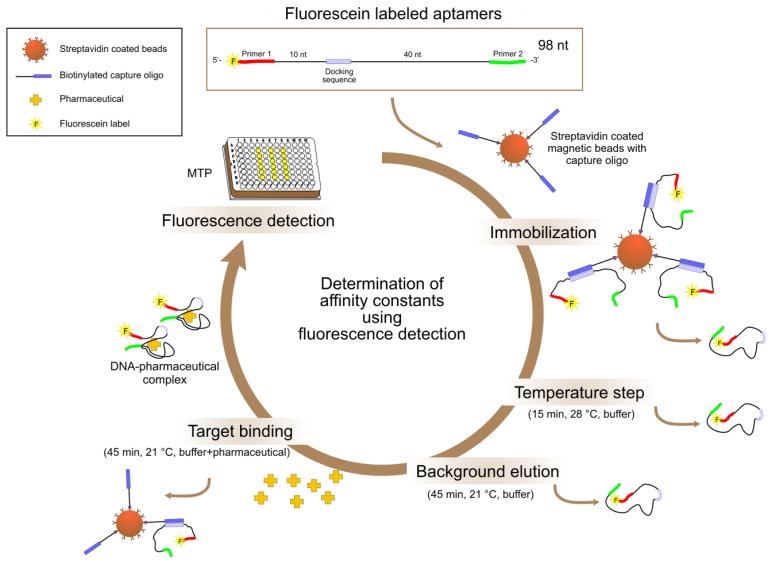
Schematic representation of the bead based assay using fluorescence detection. Fluorescein labeled aptamers: Depicted exemplary is a full length aptamer with 98 nucleobases displaying both primer binding sites. In case of deletions of single nucleobases during selection or in case of the use of truncated versions of the aptamers, the sequences are shorter.

Specificity tests by fluorescence detection in the bead based assay (see Figure 3) were performed very similar to a typical Capture-SELEX round. The 5'-FAM labeled aptamers were immobilized on magnetic beads. Target binding was measured by adding the targets in solution, incubation for binding of aptamers (with concurrent release from the bead) to the targets and fluorescence measurement in the supernatant after removal of the beads.

Firstly, an appropriate amount of streptavidin coated superparamagnetic beads were transferred from stock solution to a sample tube and washed three times with 500 μL binding and washing (B&W) buffer (10 mmol L^−1^ Tris-HCl pH 7.5, 1 mmol L^−1^ EDTA, 2 mol L^−1^ NaCl). The beads were separated from the solution by placing the tube into a magnet stand. The beads were washed and resuspended in B&W buffer to a concentration of 2 × 10^9^ beads mL^−1^. For immobilization of the capture oligonucleotides, an equal volume of the biotinylated capture oligo iODN2Sp was added (600 pmol biotinylated oligo per 1 × 10^8^ beads). This mixture was incubated at room temperature for 1 h with gentle rotation using an overhead shaker (Intelli-Mixer RM-2, neoLab, Berlin, Germany). After that, the supernatant was discarded and the beads were washed three times with 500 μL B&W buffer and afterwards three times with 500 μL selection buffer (100 mmol L^−1^ NaCl, 20 mmol L^−1^ Tris-HCl pH 7.6, 2 mmol L^−1^ MgCl_2_, 5 mmol L^−1^ KCl, 1 mmol L^−1^ CaCl_2_). After resuspension in selection buffer to a final concentration of 1 × 10^9^ beads mL^−1^, the streptavidin coated magnetic beads, now modified with the streptavidin-biotin bound biotinylated capture oligo iODN2Sp, were ready for use. The bead concentration could be determined more exactly by microscopic counting (microscope Olympus BX60, Olympus Europa Holding GmbH, Hamburg, Germany) using a Neubauer improved counting chamber.

For thermal equilibration of the aptamer ssDNA, approximately 50 pmol of oligonucleotides per 5 × 10^7^ beads were heated to 90 °C for 8 min, immediately cooled and kept at 4 °C for 10 min followed by a short incubation at room temperature. In the following, the aptamers labeled at their 5′ends with fluorescein and the beads prepared as described above were incubated together overnight with mild shaking at 21 °C. During this immobilization step, the docking sequence of the aptamers and the complementary sequence of the capture oligos hybridized and thereby the aptamers were fixed to the beads.

Unbound oligonucleotides were removed by washing the beads nine times with 500 μL selection buffer. In the temperature step all of the remaining unhybridized oligonucleotides or weakened DNA duplex structures were eliminated by incubation of the DNA-bead-complexes in 500 μL selection buffer at 28 °C for 15 min with mild shaking and subsequently washing seven times with 500 μL selection buffer. The following incubation of the DNA-bead-complexes in 300 μL selection buffer at 21 °C for 45 min with mild shaking served as control for background elution of hybridized oligonucleotides from the beads caused by the incubation procedure. After seven additional wash steps in 500 μL selection buffer, the DNA-bead-complexes were incubated at 21 °C for 45 min with mild shaking in 300 μL solution of the respective pharmaceutical to be tested with a concentration of 1 mmol L^−1^ in selection buffer. During this target binding step, oligonucleotides (aptamers) were released from the DNA bead complexes due to an affinity induced structure switch into a specific three-dimensional structure allowing binding of the aptamer to the target molecule. They could be collected in the supernatant after magnetic separation of the beads.

Fluorescence readings of the fluorescein labeled oligonucleotides were performed in black 96-well microtiter plates (NUNC) using a Wallac Multilabel Counter 1420 Victor2V (PerkinElmer Life Sciences, Freiburg, Germany) under the following conditions: prompt fluorometry, excitation filter 485 nm, emission filter 535 nm, time 1 s, CW-lamp energy 15,000. The sample volume was 100 μL per well. Calibration curves ranging from 1.9 ng mL^−1^ to 1.9 μg mL^−1^ were prepared using fluorescein labeled ssDNA of the corresponding aptamer and were subjected to the same thermal pretreatments as in the specificity tests.

##### MTP Assay

Nunc Immobilizer™ Streptavidin plates (Thermo Scientific, Roskilde, Denmark) were used for the second assay format based on fluorescence detection. Biotinylated capture oligos iODN2Sp were immobilized at an amount of 150 pmol per well on the wells of a 96 microwell plate according to the manufacturer's protocol. After washing three times with selection buffer, an amount of 10–15 pmol of thermally equilibrated aptamers labeled at their 5′ends with fluorescein was added to each well and incubated overnight at 21 °C and light agitation. After washing three times with selection buffer, different concentrations of kanamycin A in selection buffer or in a mixture of real sample and selection buffer were added to the wells and incubated 45 min at 21 °C. For the six-fold determination of one sample of distinct concentration of kanamycin A, six wells of the microplate were used. During this step the target binding takes place where the aptamers that are able to bind to the target molecule undergo a conformational change and leave the duplex with the capture oligo in order to bind to the target. Afterwards, the plate was again washed three times with selection buffer and the amount of the remaining aptamer ssDNA in the wells of the microplate was detected by fluorescence reading. Fluorescence readings of the fluorescein labeled oligonucleotides were performed as described above and data of the multiple determinations were averaged. The amount of eluted aptamers could be calculated as difference between the fluorescence counts before and after target binding by referring it to a calibration curve. The plate can be regenerated by washing it twice with 100 μL of selection buffer per well for 10 min at 50 °C with mild shaking to remove all aptamers.

#### 2.3.2. SPR Detection

**Figure 4.**
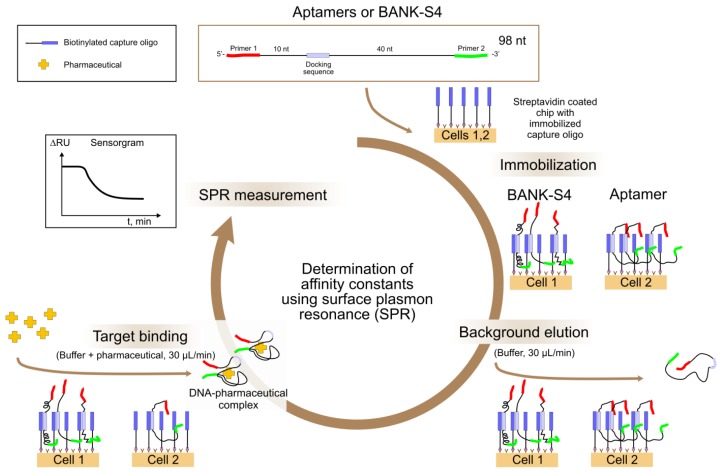
Methodology of the affinity tests performed by SPR detection. Aptamers: Depicted exemplary is a full length aptamer with 98 nucleobases displaying both primer binding sites. In case of deletions of single nucleobases during selection or in case of the use of truncated versions of the aptamers, the sequences are shorter.

In addition to specificity tests using fluorescence detection, affinity tests were performed using a surface plasmon resonance (SPR) system (Biacore X 100, GE Healthcare), see Figure 4. The surface plasmon resonance measurement is a very sensitive label free detection method which allows real time and quantitative monitoring of binding events at a sensor surface. In our case, aptamers were immobilized via capture oligo at the sensor surface and the release of the aptamers caused by binding to the target added was measured.

The Biacore X 100 system offers two flow cells in parallel within the microfluidic cartridge. The signals of the sample (cell 2) can be referenced to those of the reference (cell 1). The surface of streptavidin coated sensor chips (SA sensor chips, GE Healthcare) was firstly conditioned by washing three times with 1 mol L^−1^ NaCl, 50 mmol L^−1^ NaOH, for 1 min at a flow rate of 10 μL min ^-1^. Then biotinylated capture oligo iODN2Sp was immobilized on both, the sample and reference cells by injecting a solution of iODN2Sp at a concentration of 1 pmol μL^−1^ in selection buffer for 18 min at a flow rate of 5 mL min^−1^. The shift of the resonance angle due to the immobilized amounts of capture oligo was ∼1400 RU each. Then, at (sample) cell 2, the aptamer to be tested was immobilized via hybridization to the sequence of the capture oligo that was complementary to the docking sequence included in the aptamer sequence. For this, the aptamer was injected at a concentration of 1 pmol μL^−1^ and at a flow rate of 5 μL min^−1^ for 15 min. This led to a shift in resonance angle of 440 RU on average. In the same way, sequences of the random library BANK-S4 were immobilized on the reference cell (cell 1) which led to a shift in resonance angle of 270 RU on average. Both cells were washed with selection buffer at a flow rate of 30 μL min^−1^ for 3 min in order to remove loosely bound sequences. After that, target in increasing concentrations was injected into both cells at a flow rate of 30 μL min^−1^ for 3 min. Measured is the difference signal (cell 2–cell 1) which gives a concentration dependent negative saturation curve, caused by the target molecule binding to the aptamers by conformational change and de-hybridization of their binding to the capture oligos. In contrast, the control, BANK-S4, was hardly released and remained at the surface. Each injection of target was directly followed by an injection of selection buffer into both cells at a flow rate of 30 μL min^−1^ for 3 min. 20 s after the beginning of this second injection the current RU value was recorded, the so-called “stability” report point. The “stability” report points for each concentration were used for the calculation of the affinity constants and were fitted by the model of one site direct binding using a rectangular hyperbola for the saturation curve (OriginPro 8G SR2, OriginLab Corporation, Northampton, MA, USA).

### 2.4. Kanamycin A Detection in Real Samples

For the detection of kanamycin A in cleaned waste water, a water sample was taken in January 2013 at the drain of a vertical flow type of constructed wetlands and was experimentally spiked with the pharmaceutical. The real water was filtered by using a vacuum filtration unit (0.22 μm, Sartorius, Göttingen, Germany) and subsequently mixed in equal parts with selection buffer that was beforehand spiked with different amounts of kanamycin A. The pH of the resulting solution was about 7.6. This solution was the measuring sample added in the MTP assay (see description in Section 2.4.1) to the wells. All other steps, like washing steps and fluorescence detection, were performed in selection buffer.

## 3. Results and Discussion

### 3.1. Primary and Secondary Structures of Aptamers and Their Truncations

Considering the primary sequences in Table 1 it should be noted that the original docking sequence (TGAGGCTCGATC, highlighted in blue) of aptamers # 3_19 and # 3_7 is altered during the Capture-SELEX process compared to the originally designed sequence. This is most likely a concession to the design of the SELEX procedure as there is a selection pressure towards docking sequences that are less stably attached to the capture oligos as described in detail in [12]. For the analysis of the secondary structures of the six aptamers chosen from [12] and the secondary structures of their truncations the free-energy minimization algorithm using the web based tool mfold was used (Figure 2).

All aptamers and truncations display secondary structures that show stem-loop structures separated by unstructured regions. In case of the full length aptamers, they have four stem-loops, the truncated version # 3_7(1-79) has three stem-loops and # 13_82(19-80) has two stem-loops. One feature that all aptamers derived from this Capture-SELEX process have in common is that the docking sequence, i.e., the region complementary to the capture sequence of the capture oligo, is incorporated in a stem-loop structure. The aptamer takes on this secondary structure when free in solution. Nutiu and Li [16] firstly proposed the concept of switching of the aptamer from an oligonucleotide binding partner to the target molecule by alteration of its structure as the presence of the target causes adaptive folding of the aptamer [17]. In the end, the formation of the aptamer target complex has to be energetically more stable than the complex of aptamer and capture oligo as only then the addition of the target to a solution containing the duplex of aptamer and oligonucleotide binding partner will force the release of the aptamer from its oligonucleotide binding partner [18]. This means that structure switching aptamers only rearrange into an active target-bound structure upon binding to their target. In the absence of the target, however, they are in a less ordered state and are able to partially hybridize with a complementary oligonucleotide [17].

When looking at the truncated versions where the 3′or 5′or both primer binding sites of the tested aptamers # 3_7, # 13_82, # 11_76, and # 3_19 were cut, it can be seen that some of these truncated versions still show binding activity.

For some aptamers the primer binding sites, even as they displayed fixed sequences and were designed beforehand, are necessary for binding. This is the case for aptamer # 3_19, where not one of the truncated versions of the aptamer were able to bind to the target kanamycin A and for aptamer # 11_76, where only the version with truncated 5′primer binding site (# 11_76(1-79)) displayed binding to kanamycin A, but to a much lesser extent than the parental aptamer (80 percent of the value of the parental aptamer). In contrast, truncation of the aptamers # 3_7 and # 13_82 influences the binding to kanamycin A not (# 13_82) or even leads to an enhancement of the binding (# 3_7(1-79), enhancement by 225 percent of the value of the parental aptamer).

The therapeutic mode of action of aminoglycoside antibiotics is that they bind to RNA, namely the 16 S RNA of ribosomes, and thereby hinder translation. The binding motif at the RNA side is a stem-loop structure [19–21]. It was shown that artificial RNA aptamers for aminoglycoside antibiotics also display a stem-loop structure as binding site forming pockets for the aminoglycoside antibiotics [22]. Although RNA and DNA are different in their binding behaviour, also in our case of DNA aptamers, there is a strong possibility that the binding region is a strem-loop structure as this conformation is adopted by the aptamers when not hybridized to the capture oligo. However, for some aptamers the other nucleobases also play an important role in target binding as even the primer binding sites are necessary for successful recognition of the target molecule, as was stated above. More detailed analysis will be necessary in order to determine the exact binding motif.

### 3.2. Specificities of the Derived Aptamers to Different Aminoglycoside Antibiotics

Specificities of the derived aptamers to different aminoglycoside antibiotics (see Figure 1b) were determined by use of the bead assay which was performed similar to the SELEX-procedure. Additionally binding of the aptamers to the amino sugars glucosamine and N-acetyl-D-glucosamine (see Figure 1c) was tested. Aminoglycoside antibiotics were chosen from very similar to the selection target kanamycin A (kanamycin B which differs only in one functional group and tobramycin that also belongs to the kanamycin subgroup of aminoglycoside antibiotics and has pyranose sugars attached to the 4 and 6 positions of the cyclohexyl-2-deoxistreptamidine ring [23]) to those that display similarity to the selection target to a much lesser extent, for example neomycin, which together with paromomycin belongs to a separate subgroup of aminoglycoside antibiotics and has a pyranose sugar attached to the 4 position and a furanose-pyranose pair of sugars attached to the 5 position of the 2-deoxystreptamidine ring [23].

The aim was to test the variety of binding among the different aptamers. Additionally, as aminoglycoside antibiotics are composed of amino sugars, it was tested whether the aptamers for kanamycin A bind those sugars as well.

It could be seen that aptamer # 3_19 was the most specific among all aptamers tested. Except kanamycin A only apramycin could evoke an elution of aptamer DNA above the threshold of 1 pmol eluted ssDNA when the pharmaceuticals were added with a concentration of 1 mmol L^−1^ in selection buffer. For kanamycin A, the amount of eluted aptamer DNA was 3.02 pmol, for apramycin 2.27 pmol. The truncated aptamer # 13_82(19-80) was eluted by kanamycin A (6.87 pmol), kanamycin B (3.09 pmol), and tobramycin (1.13 pmol) whereas its parental aptamer, aptamer # 13_82 had a broader spectrum of binding aminoglycoside antibiotics: Besides binding to kanamycin A (6.85 pmol) it bound to kanamycin B (7.22 pmol), apramycin (3.40 pmol), tobramycin (2.78 pmol), and netilmicin (1.01 pmol). The broadest spectrum of binding aminoglycoside antibiotics had the aptamer # 11_76. Apart from kanamycin A (5.02 pmol) and kanamycin B (5.21 pmol) it was also eluted by tobramycin (4.03 pmol), apramycin (3.04 pmol) and, to a lesser extent, by gentamicin (2.00 pmol) and netilmicin (1.03 pmol). The aptamer with the highest values of binding given as eluted DNA was the truncated aptamer # 3_7(1-79). At 1 mmol L^−1^ concentration, kanamycin A eluted 10.44 pmol aptamer DNA, kanamycin B 8.04 pmol, whereas apramycin (1.68 pmol) and tobramycin (1.91 pmol) eluted this aptamer to a much lesser extent. Its parental aptamer, # 3_7 was eluted by kanamycin A (4.62 pmol), kanamycin B (4.42 pmol), apramycin (3.04 pmol), and netilmicin (1.03 pmol).

### 3.3. Epitope-like Regions on Pharmaceuticals

When looking at the binding complex of DNA aptamer and aminoglycoside antibiotic, especially kanamycin A as the target molecule from the point of view of the antibiotic there are possible explanations for the differences in binding specificities as described above. From the literature it could be seen that for the binding of RNA aptamers developed for the detection of tobramycin, intermolecular hydrogen bonding between charged NH_3_^+^ groups of the antibiotic and acceptor atoms of the nucleobases of the aptamer was crucial [23]. For the two RNA aptamers for tobramycin compared in that review, one exhibited binding to rings I and III, the other to rings I and II of the antibiotic. As in our case the aptamer consisted of DNA in contrast to RNA examined in the before-mentioned study, one cannot expect an identical binding region within the aptamer. Nevertheless it is possible by regarding the different binding patterns of the aptamers to the very closely related antibiotics kanamycin A, as well as kanamycin B, and tobramycin that differ from kanamycin A only in one and two functional groups at ring I, respectively (see Figure 1). At a pH of about 7.6 that was used throughout in our studies, only the amino groups at the 5 position of ring I of kanamycin A and at the 2 position of ring I of kanamycin B and tobramycin were present in the charged form of NH_3_^+^ [13]. As aptamer # 3_19 only binds to kanamycin A when comparing these closely related compounds it is very probable that binding involves ring I of the pharmaceutical. In contrast, regarding aptamer # 11_76 that binds all three antibiotics nearly to the same degree, ring I will probably not play such a crucial role. For aptamers # 3_7, # 3_7(1-79) and # 13_82 binding to kanamycin A and B are nearly identical, whereas binding to tobramycin is less strong. Therefore one could assume that the presence of the hydroxyl group at the 3 position of ring I here is crucial for binding. Regarding aptamer # 13_82(19-80) that shows binding to kanamycin A, B and tobramycin in decreasing ranges both functional groups that differ seem to be involved in binding. For a more detailed view into the binding mechanisms it would be desirable to perform high resolution studies, like NMR.

### 3.4. Dissociation Constants Obtained by Fluorescence and SPR Detections for Aptamers and Their Truncations

**Figure 5.**
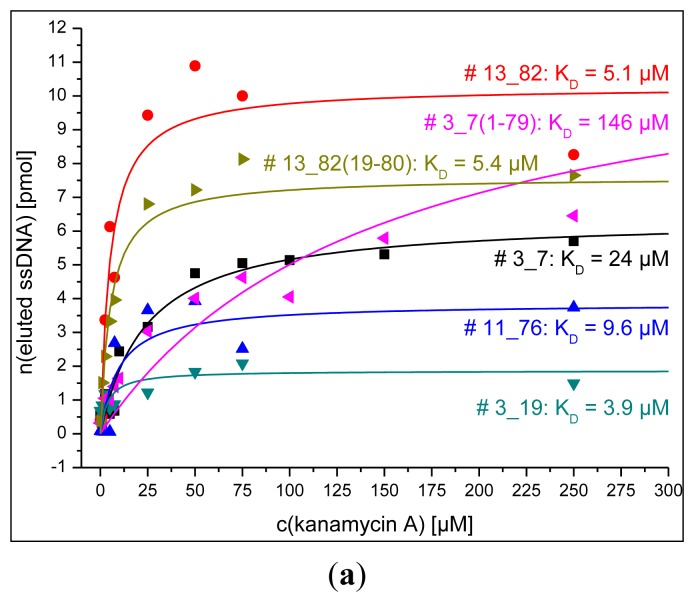

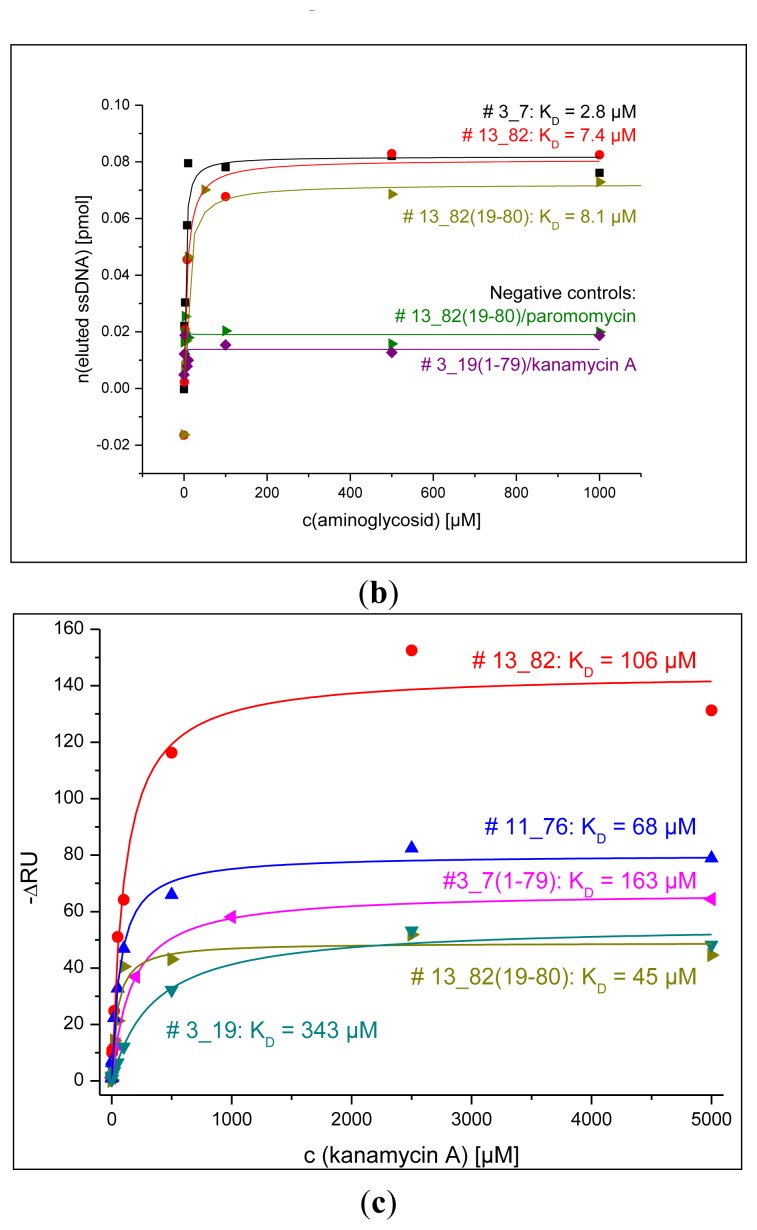
(**a**) Determination of dissociation constants by fluorescence detection using the bead assay; (**b**) by fluorescence detection using the MTP assay also showing two negative controls (aptamer truncations # 13_82(19-80) tested with paromomycin that is not bound and # 3_19(1-79), which lost binding properties due to truncation, together with kanamycin A; and (**c**) by SPR detection using Biacore X 100 (GE Healthcare).

Figure 5 shows the results from the determination of dissociation constants obtained by fluorescence measurement using the bead assay or MTP assay and SPR detection as described in the experimental section. All dissociation constants obtained by these techniques are in the micromolar range for all tested aptamers and their truncations.

Song *et al.* developed a DNA aptamer for kanamycin using affinity chromatography with kanamycin-immobilized sepharose beads which exhibited affinities for kanamycin A, B, and tobramycin in the nanomolar range [24]. The RNA aptamers for aminoglycoside antibiotics developed by Goertz *et al.* using an automated selection protocol have dissociation constants that also lie in the nanomolar range [25]. Kwon *et al.* found RNA aptamers for kanamycin B that showed high nanomolar affinity to the targeted aminoglycoside using surface plasmon resonance and fluorescence anisotropy techniques [26].

Two negative controls using the MTP assay were conducted and consisted of the combination of a binding aptamer for kanamycin A (aptamer # 13_82(19-80)) and an aminoglycoside antibiotic that is not detected by this aptamer (paromomycin) and of the combination of an aptamer that lost its binding properties by truncation (sequence # 3_19(1-79)) and kanamycin A. Both setups showed elution of ssDNA that did not exceed background elution (*cf*. Figure 5b).

### 3.5. Kanamycin A Detection in Real Waste Water Sample

The aim of this part of the work was to detect kanamycin A in a real water sample on the way to a robust and functional assay that can be used for water analysis. For that purpose, an experimentally spiked water sample of treated sewage effluent from a vertical flow type of constructed wetlands was analysed using the MTP assay as described in section 2.4.1—MTP Assay and section 2.5. Aptamer # 3_7 and kanamycin A in different concentrations ranging from 0 to 500 μmol L^−1^ in the solution that consisted of the real sample mixed with selection buffer in equal parts were used. The detection limit, defined as a signal-to-noise ratio of 3, was 5 μmol L^−1^ kanamycin A and the measuring range was 0 to 50 μmol L^−1^ kanamycin A in the measuring solution.

## 4. Conclusions/Outlook

With this work we could show that aptamers (and some truncations thereof) that were derived from a Capture-SELEX procedure which targeted against kanamycin A displayed binding to other aminoglycoside antibiotics as well. The binding patterns differed among all tested aptamers so that there were highly substance specific aptamers (such as aptamer # 3_19, binding only to kanamycin A and apramycin) and more group specific aptamers binding to a different variety of aminoglycoside antibiotics. The epitope-like region of the aminoglycoside antibiotics responsible for aptamer binding could be defined. Affinities of the different aptamers for their target substance, kanamycin A, could be measured with different approaches and were in the micromolar range. Finally, the proof of principle of an aptamer based assay allowed the detection of kanamycin A in a real water sample could be given.

Future work will comprise a further isolation of the epitope-like region in the aminoglycoside antibiotics or even the visualization with imaging methods. The aptamer based assay for the detection of kanamycin A or other aminoglycoside antibiotics in environmental samples will be modified in order to get higher output values or the detection principle will be altered in order to achieve that goal. Aptamers that show group specificity to aminoglycoside antibiotics can be used as novel filter material for the elimination of those pharmaceutical or their residues from environment samples.

## Figures and Tables

**Table 1.**
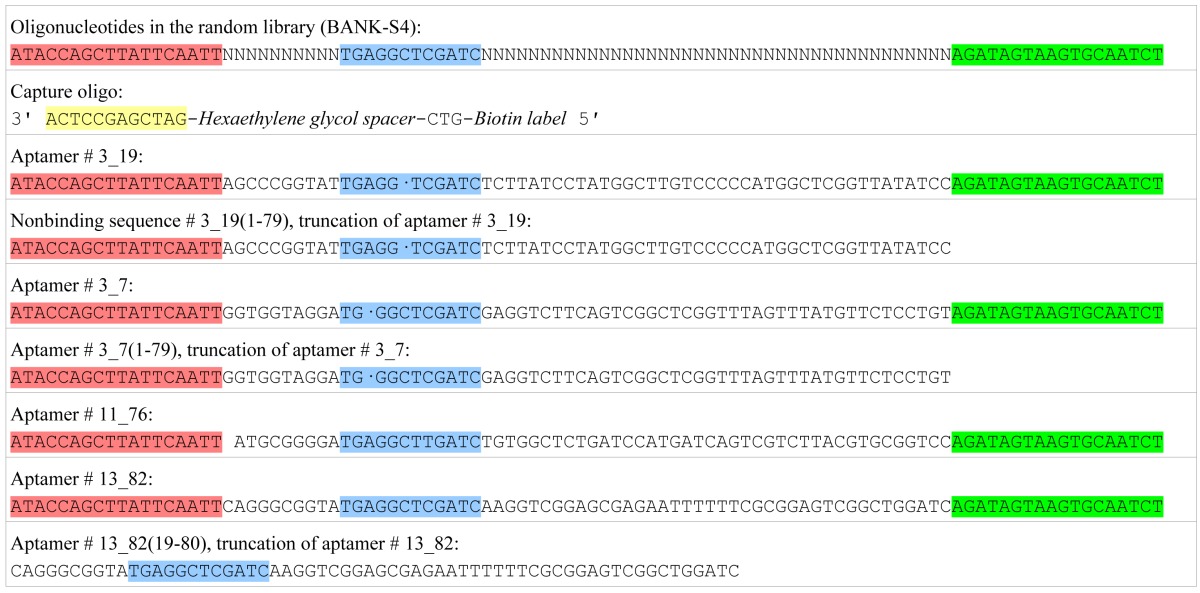
Sequences of the oligonucleotides in the random library, of the capture oligo, aptamers for kanamycin A, derived from Capture-SELEX [12], and truncations thereof. The 5′and 3′primer binding sites are highlighted in red and green, respectively. The docking sequence is highlighted in blue and its complementary part in the capture oligo is highlighted in yellow. Deletions in the sequence compared to the starting random library are denoted as a dot. Direction is 5'→3′if not stated otherwise.
